# Experimental capture of miRNA targetomes: disease-specific 3′UTR library-based miRNA targetomics for Parkinson’s disease

**DOI:** 10.1038/s12276-024-01202-5

**Published:** 2024-04-01

**Authors:** Martin Hart, Fabian Kern, Claudia Fecher-Trost, Lena Krammes, Ernesto Aparicio, Annika Engel, Pascal Hirsch, Viktoria Wagner, Verena Keller, Georges Pierre Schmartz, Stefanie Rheinheimer, Caroline Diener, Ulrike Fischer, Jens Mayer, Markus R. Meyer, Veit Flockerzi, Andreas Keller, Eckart Meese

**Affiliations:** 1 Human Genetics, Saarland University, 66421 Homburg, Germany; 2 Clinical Bioinformatics, Saarland University, 66123 Saarbrücken, Germany; 3 Helmholtz Institute for Pharmaceutical Research Saarland (HIPS)–Helmholtz Centre for Infection Research (HZI), Saarland University Campus, Saarbrücken, Germany; 4 Department of Experimental and Clinical Pharmacology & Toxicology, Institute of Experimental and Clinical Pharmacology and Toxicology, Center for Molecular Signaling (PZMS), Saarland University, 66421 Homburg, Germany; 5 Department for Internal Medicine II, Saarland University Hospital, 66421 Homburg, Germany

**Keywords:** miRNAs, Cell signalling, Parkinson's disease

## Abstract

The identification of targetomes remains a challenge given the pleiotropic effect of miRNAs, the limited effects of miRNAs on individual targets, and the sheer number of estimated miRNA–target gene interactions (MTIs), which is around 44,571,700. Currently, targetome identification for single miRNAs relies on computational evidence and functional studies covering smaller numbers of targets. To ensure that the targetome analysis could be experimentally verified by functional assays, we employed a systematic approach and explored the targetomes of four miRNAs (miR-129-5p, miR-129-1-3p, miR-133b, and miR-873-5p) by analyzing 410 predicted target genes, both of which were previously associated with Parkinson’s disease (PD). After performing 13,536 transfections, we validated 442 of the 705 putative MTIs (62,7%) through dual luciferase reporter assays. These analyses increased the number of validated MTIs by at least 2.1-fold for miR-133b and by a maximum of 24.3-fold for miR-873-5p. Our study contributes to the experimental capture of miRNA targetomes by addressing i) the ratio of experimentally verified MTIs to predicted MTIs, ii) the sizes of disease-related miRNA targetomes, and iii) the density of MTI networks. A web service to support the analyses on the MTI level is available online (https://ccb-web.cs.uni-saarland.de/utr-seremato), and all the data have been added to the miRATBase database (https://ccb-web.cs.uni-saarland.de/miratbase).

## New Approach Boosts Parkinson’s Disease-Related miRNA Target Identification

This research aimed to explore the relationship between microRNAs and target genes, key to understanding molecular signals in diseases like Parkinson’s disease. The scientists used a method involving a collection of resources to identify potential interactions between microRNAs and their target genes in PD. They chose four microRNAs linked with PD and found 705 potential interactions. Using a partly automated test, they confirmed 442 of these interactions. The research also discovered that the effectiveness of microRNAs differs among various signal pathways. The results suggest that the local secondary structure of the miRNA binding site within the 3’ untranslated region of the target gene has a limited effect on the tests. This study lays the groundwork for further testing of potential interactions in specific disease characteristics. The results could impact the creation of microRNA-based treatments. This summary was initially drafted using artificial intelligence, then revised and fact-checked by the author.

## Introduction

miRNAs posttranscriptionally regulate the expression of target genes mainly by binding to the 3′ untranslated region (3′UTR) and rarely to the 5′UTR or the open reading frame of their target genes^1–3^. Depending on the complementarity of the miRNA seed region and the corresponding 3′UTR sequence of the target genes, posttranscriptional regulation can lead to mRNA destabilization, degradation, or inhibition of protein translation^4–7^. Therefore, miRNAs regulate many target genes and are associated with a variety of cellular processes and different human diseases^8–10^. Although hundreds to thousands of papers connect miRNAs to genes involved in diseases, the basis for this link, (i.e., evidence of a miRNA targeting a gene and affecting a given disease), is often missing or purely descriptive. Recently, miRNAs have been proposed to be powerful regulators of ribosome biogenesis, suggesting new applications for microRNA-mimic chemotherapeutics^11^. Consequently, miRNAs have been identified as biomarkers and candidates for these and other therapeutic approaches^12–15^. However, incorporating miRNAs in different therapeutic strategies requires the best possible knowledge of their target genes to avoid adverse side effects^16,17^. Notably, the exact mechanism through which miRNAs target genes is not fully understood. For example, studies suggest that argonaute binding within 3′-untranslated regions poorly predicts gene repression^18^.

Unfortunately, the full spectrum of miRNA–target gene interactions (MTIs) has not been fully elucidated due to the sheer number of more than 40 million MTIs^19^. This number also explains why knowledge about MTIs is mostly based on prediction algorithms that frequently yield heterogeneous and partially unspecific results^20^. Approaches to providing experimental evidence for MTIs are frequently only descriptive and complicated by the pleiotropic effect of each miRNA and by rather limited effects on each single target^21^. Ideally, functional assays would take into account the different cellular backgrounds since MTIs are likely specific to tissues, cell types and disease states^22^. This, however, complicates the task of collecting further experimental evidence for MTIs since the potential MTIs would each have to be tested in specific cellular contexts. Although high-throughput methods such as CLIP-seq (cross-linking immunoprecipitation-high-throughput sequencing) or CLASH (cross-linking, ligation, and sequencing of hybrids) have the power to identify an enormous number of potential MTIs, they cannot discriminate between a functional MTI causing the downregulation of the target gene at the protein level and a short binding of the miRNA to its target followed by the release of mRNA and miRNA from the RISC without any functional relevance. Here, we aimed to validate the complete target spectra of specific miRNAs under standardized conditions in a specific disease context. Since miRNAs have increasingly gained attention in Parkinson’s disease (PD) research, we selected this disease, which is the second most common neurodegenerative disorder, to test our targetome capturing technique^23–25^.

**Fig. 1:**
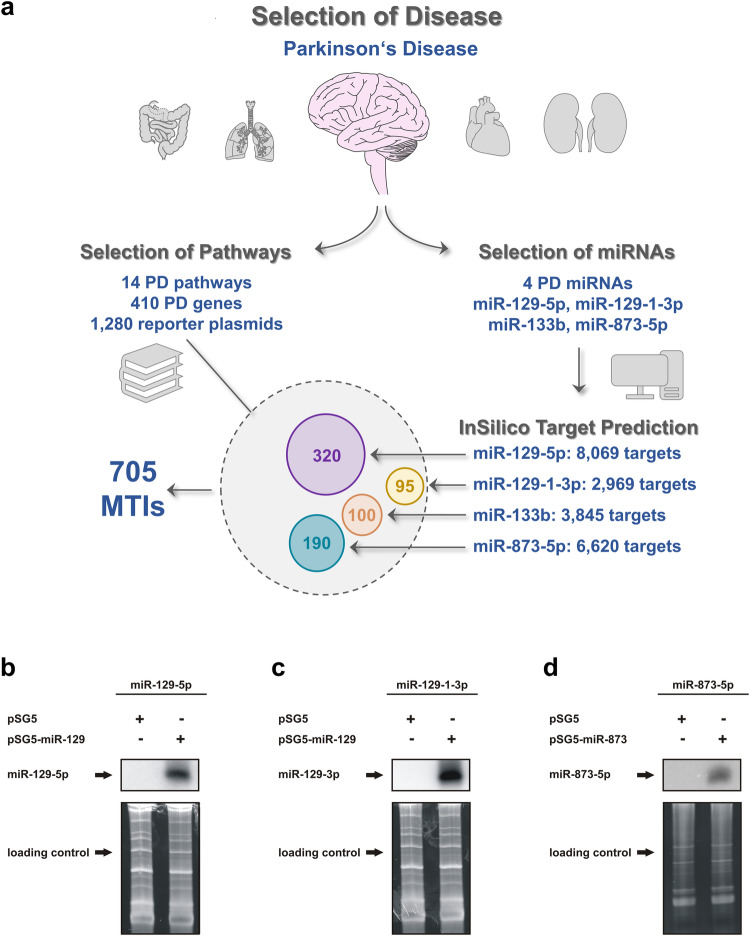
Library-based miRNA target gene reporter assay. **a** Selection of MTIs. The MTIs were defined in a staged concept. Using PD as the disease phenotype, we collected data on 14 PD pathways involving 410 genes. From these genes, 1280 3′UTR reporter constructs were derived considering length constraints. We selected four relevant PD miRNAs unrelated to the analyzed genes. Via computational prediction, for the miR-129-5p 320 3′UTR reporter construct, 95 3′UTR constructs were used for miR-129-1-3p, 100 3′UTR constructs were used for miR-133b, and 190 3′UTR constructs were identified for miR-873-5p harboring at least one canonical binding site on the respective miRNA. In total, 705 MTIs for the four chosen miRNAs were analyzed. The 3′UTRs of the target genes harboring more than one exclusive miRNA binding site are shown in Supplementary Table 7, and the overlaps are visualized in Supplementary Fig. 1. **b** Northern blot analysis of miR-129-5p. 293 T cells were transfected with miRNA expression plasmids containing the sequence of miR-129-5p, miR-129-1-3p, or miR-873-5p. After 48 h, RNA was isolated, and Northern blot analysis was performed with specific probes against the indicated miRNAs. **c** Northern blot analysis of miR-129-1-3p. 293T cells were transfected with a miRNA expression plasmid containing the sequence of miR-129-1-3p. After 48 h, RNA was isolated, and Northern blot analysis was performed with specific probes against miR-129-1-3p. **d** Northern blot analysis of miR-873-5p. 293 T cells were transfected with a miRNA expression plasmid containing the sequence of miR-873-5p. After 48 h, RNA was isolated, and Northern blot analysis was performed with specific probes against miR-873-5p.

We calculated a maximum of 44,571,700 miRNA–target gene interactions (MTIs) based on 19,379 protein-coding human genes (GENCODE Release version 42) and an estimated number of 2300 human miRNAs^23^. This calculation does not acknowledge the possibility of obtaining different transcripts from single genes or from different tissue- and cell type-specific MTIs. Since the first reviews on methods for miRNA target validation in 2008^21^, reporter assays are still considered the primary method for functionally analyzing the binding of a miRNA to a single target. However, the efficacy of this method is limited. Even in a simplified scenario with a single miRNA tested for all its predicted interactions to human protein-coding genes, our semiautomated high-throughput miRNA interaction reporter assay (HiTmIR) as it is used in this study enables at maximum the analysis of 120 3′UTR sequences within two weeks (assuming 20 target UTRs per 96-well plate, at an analysis time of 12 plates per week, and four replicates). Hence, the analysis of all 8,069 highly confidently predicted MTIs for miR-129-5p would take more than two years. To narrow the scope of this task, we limited our analysis to Parkinson’s disease, as described above^26,27^ (Fig. 1a). Notably, we selected the miRNAs and the mRNAs based only on their association with PD without considering whether the mRNAs were predicted as targets of the chosen miRNAs. Since there are no publicly available human 3′UTR libraries that are enriched for genes belonging to a given disease phenotype and/or specific biological pathway, we generated a 3′UTR library that contained 410 potential miRNA target genes, all of which were reported to be involved in signaling pathways associated with PD.

In detail, these pathways included 14 PD- and dopamine-associated signaling pathways, including the signaling pathway dopaminergic synapse (KEGG, hsa04728), dopamine metabolic process (Gene Ontology, GO:0042417), and dopaminergic neurogenesis (WikiPathways, WP2855). We synthetized the 3′UTRs of the 410 genes as approx. 675 bp long fragments to account for length restrictions of the reporter gene plasmids, as addressed in our previous studies^23^. The fragments were designed with a 30 bp overlap between consecutive fragments of a 3′UTR. The resulting library with 1,280 3′UTR sequences cloned in the reporter plasmid pMIR-RNL-TK (Supplementary Table 1) enables target screening of any miRNA. With this study, we aimed to validate the disease-specific targetomes of four miRNAs. Using a library of 3′UTRs specific to PD allowed us to assess the effect of disease-associated miRNAs on the regulation of key pathological players, enabling the elucidation of disease-associated miRNA‒target‒gene networks. The advantages of this method include flexibility in testing numerous disease-associated miRNAs; reproducibility, reliability and comparability of results due to the use of a standardized test method with identical 3′UTR sequences; and the possibility of further investigating the fundamental mechanisms of synergistic miR targeting in future studies.

## Materials and methods

### Cell lines

The cell lines HEK 293 T (ACC 635) and SH-SY5Y (ACC 209) were purchased from the German Collection of Microorganisms and Cell Cultures (DSMZ). The authenticity of the cell lines was confirmed by short tandem repeat (STR) fingerprinting by the supplier. The cell lines were cultivated in DMEM (Life Technologies, Darmstadt, Germany) supplemented with penicillin (100 U/ml), streptomycin (100 µg/ml) and either 10% [v/v] FCS for 293T cells or 20% [v/v] FCS for SH-SY5Y cells. Subculturing was performed two times a week for no more than three months after the start of culture.

### miRNA expression plasmids

Sequence inserts representing miRNA precursor sequences with additional nucleotides up- and downstream of the precursor sequence for miR-129, miR-133b, and miR-873 were either synthesized by Eurofins Genomics (Ebersberg, Germany) or amplified by PCR using sequence-specific primers with human genomic DNA as a template and subsequently cloned and inserted into the expression plasmid pSG5 (Agilent Technologies, Santa Clara, California) using the EcoR I and BamH I restriction sites. The expression plasmid pSG5-miR-129 harbors the nucleotides 128207756-128208053[+] of human chromosome 7 (GRCh38/hg38), the pSG5-miR-133b nucleotides 52148873-52149091[+] of human chromosome 6 (GRCh38/hg38), and the pSG5-miR-873 nucleotides 28888786-28889058[-] of human chromosome 9 (GRCh38/hg38). Cloned sequences of the miRNA expression plasmids were verified by Sanger sequencing. Cloning of pSG5-miR-133b was performed as described in our previous study^19^.

### 3′UTR reporter plasmids

As positive controls for regulatory effects, sequence inserts containing two copies of the complementary sequence for the respective miRNAs as well as additional random flanking nucleotides with no self-complementary sites were generated in silico. Respective sequences were synthesized by Eurofins Genomics (Ebersberg, Germany) and cloned and inserted into the reporter plasmid pMIR-RNL-TK using the SpeI and SacI restriction sites for the positive controls of miR-129-5p, miR-129-3p, and miR-873-5p. Cloning of the pMIR-miR-133b positive control was performed employing the SpeI and NaeI restriction sites. Reporter plasmids of the PD-specific 3′UTR library containing approx. 675 bp long fragments of 3′UTR sequences of PD-associated target genes were synthesized and cloned and inserted into the reporter plasmid pMIR-RNL-TK employing SpeI and SacI restriction sites. The synthesis of 28 3′UTR sequence fragments failed. The remaining 1,280 3′UTR sequences were cloned and inserted into the reporter plasmid pMIR-RNL-TK, and the correct cloning of all reporter constructs was verified by Sanger sequencing. A complete list of all 3′UTR sequences of the PD-specific 3′UTR library, including the respective NM accession numbers, is given in Supplementary Table 1.

### Northern blot analysis

Ectopic miRNA expression and correct miRNA processing in 293 T cells were verified by Northern blotting. To do so, 293 T cells were seeded at 2.5 × 10^5^ cells per well in a 6-well plate. After 24 h, the cells were transfected with 2 µg of empty pSG5 plasmid or 2 µg of miRNA expression plasmid using Polyfect transfection reagent according to the manufacturer’s instructions (Qiagen, Hilden, Germany). After an additional 48 h, the cells were lysed using QiAzol Lysis Reagent (Qiagen, Hilden, Germany). Total RNA was isolated using a miRNeasy Mini Kit following the manufacturer’s protocol (Qiagen, Hilden, Germany). Northern blotting was performed employing radiolabeled DNA probes specific for miR-129-5p (5′CTTTTTGCGGTCTGGGCTTGCCCTGTCTC3′), miR-129-1-3p (5′AAGCCCTTACCCCAAAAAGTATCCTGTCTC3′), and miR-873-5p (5′GCAGGAACTTGTGAGTCTCCTCCTGTCTC3′) as described previously^19^.

### Library-based miRNA target gene reporter-assay (LiMTaR)

To construct the PD-specific 3′UTR library, we selected 416 target genes from 14 PD-associated pathways from the Kyoto Encyclopedia of Genes and Genomes (KEGG)^28^, Gene Ontology (GO)^29^, WikiPathways^30^ and Reactome^31^ databases. The 3′UTR sequences of the respective target genes were split into approximately 675 bp fragments, with an overlap of 30 bp between consecutive fragments. The respective sequences were synthesized and cloned and inserted into pMIR-RNL-TK. Twenty-eight 3′UTR sequence fragments were excluded due to critical sequence motifs that prevented correct synthesis. In total, we generated a PD-specific 3′UTR library that included 1,280 reporter plasmids containing 3′UTR sequences of the 410 PD-associated genes. To test the impact of the miRNAs miR-129-5p, miR-129-1-3p, miR-133b and miR-873-5p on PD-related genes, we selected the corresponding 3′UTR reporter plasmids harboring the respective binding site(s) for each miRNA from the 3′UTR library. To verify the validity of the HITmiR-Assay, each 96-well plate contained 2 positive controls (sensor reporter plasmids harboring 2 sequences complementary to the respective miRNA) at various positions to exclude positioning effects. The results of these positive controls are depicted in Supplementary Fig. 1. High-throughput analysis of reporter constructs of our PD-specific 3′UTR library was performed by a liquid handling system as described by our group previously^23^. In brief, 293T cells were seeded at 3.2 × 10^4^ cells per well in a 96-well plate using the liquid handling system epMotion® 5075 (Eppendorf, Hamburg, Germany). After 24 h, the cells were transfected with 50 ng/well of either the reporter plasmid pMIR-RNL-TK, with or without an insert, and 200 ng/well of the miRNA expression plasmid containing either the respective miRNA or no insert with PolyFect transfection reagent (Qiagen, Hilden, Germany). After an additional 48 h, the cells were lysed in passive lysis buffer (Promega, Madison, WI, USA). Luciferase substrates from the Dual-Luciferase® Reporter Assay System (Promega, Madison, WI, USA) were added to the cell lysates, and luciferase activity was measured using a GloMax Navigator microplate luminometer (Promega, Madison, WI, USA). High-throughput dual-luciferase assays were performed four times in technical duplicates. Statistical significance was calculated by Welch’s *t* test in GraphPad Prism 9.

### Quantitative real-time PCR

Ectopic expression of miR-129-5p in SHSY5Y cells was verified by quantitative real-time PCR (Supplementary Fig. 2). SH-SY5Y cells were seeded at 4.5 ×10^5^ cells per well in a 6-well plate. After 24 h, the cells were transfected with either Allstars Negative Control (ANC; Qiagen, Hilden, Germany) or the miR-129-5p miScript miRNA Mimic (MIMAT0000242, sequence: 5′CUUUUUGCGGUCUGGGCUUGC3′; Qiagen, Hilden, Germany) using HiPerFect Transfection Reagent (Qiagen, Hilden, Germany). After an additional 48 h, the cells were lysed using QiAzol Lysis Reagent (Qiagen, Hilden, Germany). Total RNA was isolated using miRNeasy Mini Kit following the manufacturer’s protocol (Qiagen, Hilden, Germany). Total RNA (150 ng) was reverse transcribed using miScript II RT Kit (Qiagen, Hilden, Germany). qPCR was performed using the miScript Primer Assay (Qiagen, Hilden, Germany) with primers specific for miR-129-5p via the StepOnePlus Real-Time PCR System (Applied Biosystems, Foster City, United States). RNU6B (Qiagen, Hilden, Germany) served as an endogenous control. The statistical significance of differences among three independent replicates was calculated by Student’s t test in GraphPad Prism 9.

### Western blot analysis

For Western blot analysis, SH-SY5Y cells were transfected with the miR-129-5p mimic as described above. After 48 h, the cells were harvested, lysed in 2x sample buffer (130 mM Tris/HCl, 6% [v/v] SDS, 10% [v/v] 3-mercapto-1,2-propandiol, and 10% [v/v] glycerol) and sonicated three times for three seconds each. Total protein extract (10 µg) was electrophoresed in a 4-15% TGX gel (Bio-Rad Laboratories, Inc., Hercules, California, USA). The proteins were electroblotted onto nitrocellulose membranes (Whatman, GE Healthcare, Freiburg, Germany). Unspecific antibody binding was blocked by preincubation of the nitrocellulose membrane in 5% TBS milk with 0.1% Tween 20 for 30 min. SNCA was detected by a polyclonal rabbit antibody (#2642), COMT by a monoclonal rabbit antibody (#14368), CLOCK by a monoclonal rabbit antibody (#5157), and AKT3 by a monoclonal rabbit antibody (#14982), all of which were purchased from Cell Signaling Technology (Danvers, MA, USA). α-Tubulin served as an endogenous control and was detected by a monoclonal rabbit antibody (#2125 Cell Signaling Technology, Danvers, MA, USA). A secondary anti-rabbit antibody was purchased from Sigma‒Aldrich (A0545; Sigma Aldrich, Munich, Germany).

### Identification of miRNA binding sites in 3′UTR sequences in silico and secondary structure matching

In this analysis, we included miR-7-5p and -34a-5p from our previous study, which were also tested with our standardized high-throughput miRNA interaction reporter assay (HiTmIR) in the context of PD^23^. For miR-129-5p, miR-129-1-3p, miR-133b, miR-873-5p, miR-7-5p and miR-34a-5p, we aligned the 3′UTR sequences of the target genes with the seed sequences of the respective miRNAs, focusing on the 8-mer, 7-mer-m8, 7-mer- A1, and 6-mer binding sites. Next, we searched for exact hits of these canonical binding sites in the 3′UTR sequences for each gene. For each 3′UTR sequence, we computed the secondary structure using RNAfold^32^. Here, we computed the minimum free energy and partition function; we did not allow GU pairs at the end of the helices and avoided isolated base pairs. We matched the dot-bracket notation of the folded 3′ UTR sequence for each binding site and computed the percentage of bound bases within the local secondary structure (identified by “(“ or “)”). We then related the percentage of bases bound within each binding site to the corresponding reduction in relative light units (RLUs) that was measured for each individual MTI.

### Calculation of the coverage score of binding sites in the 3′UTRs

Using the Graph Modeling Language representation obtained from ViennaRNA (version 2.5.1), we determined a coverage value that provides information on how many bases are found in the 2D local area around the seed region. For each base in a seed sequence, a value that represents how many bases are located within a circle with a radius (r) around the seed base is assigned; this process is performed by using the Euclidean distance. To obtain the coverage value, we summed the values of the individual bases of the seed and divided this value by the number of bases in the seed to normalize seeds of different lengths. If more than one seed region was found, we always considered the seed with the smallest coverage value, corresponding to the most accessible site, for further analysis. For the calculation of correlations, we used Pearson correlation.

Formally, the coverage score is defined as follows: Let ${B}_{r}\left(x\right):=\left\{x\in {R}^{2}|\,{{||x||}}_{2}\le r\right\}$ be the 2-dimensional ball of radius $r\in {{\mathbb{R}}}_{\ge 0}$ and

$${\chi }_{A}(x):=\left\{\begin{array}{c}1,\text{if}\,x\in A\\ 0,\text{if}\,x\notin A\end{array}\right.$$

the indicator function. The RNA secondary structure of a UTR sequence is given as a graph *G* with $G=\left(V,E\right)$, where *V* denotes the vertices and *E* the edges. In our case, the vertices correspond to the bases and the edges to the molecular compounds and to the hydrogen bonds. There is an edge ${e}_{i}\in E$ between two vertices ${v}_{i}$, ${v}_{i+1}\in V$ since the two bases are next to each other in the RNA secondary structure, so the edge ${e}_{i}$ corresponds to a molecular compound. There is an edge ${e}_{k}\in E$ with *k* ≥ ${|V|}$ if there is a hydrogen bond between two vertices ${v}_{i},{v}_{j}\in V$ and $i\, <\, j$. Let ${S}_{l}\subseteq V$ be the set of vertices contained in the *l*-th seed region of a miRNA.

The *coverage score* for this miRNA, the seed region ${S}_{l}$ and a fixed $r\in {{\mathbb{R}}}_{\ge 0}$ were defined as follows:

$$\frac{\sum _{{s}_{l}\in {S}_{l}}\sum _{v\in V}{\chi }_{{B}_{r}\left({s}_{l}\right)}\left(v\right)}{|{S}_{l}|}$$

where $\sum _{v\in V}{\chi }_{{B}_{r}\left({s}_{l}\right)}\left(v\right)$ gives the number of bases in a 2-dimensional ball around the seed bases ${s}_{l}$. Therefore, the coverage score yields the sum of the number of bases in the radius of the seed bases normalized by the number of bases in the seed region.

The calculations were carried out with python with the packages pandas (version 3.10.8), numpy (1.24.2), matplotlib (3.7.0), networkX (3.0), pillow (9.4.0) and scipy (1.10.1). The package scipy with the included interpolation method and the package Pillow were used to obtain line art figures.

## Results

### Identification of 705 MTIs between the 410 genes and 4 PD-miRNAs

To demonstrate the potential of the PD-specific 3′UTR library, we explored the complex miRNA‒target interactions of four well-known PD-related miRNAs (miR-129-5p, miR-129-1-3p, miR-133b, and miR-873-5p)^23,33–35^ (Fig. 1a). Notably, we intentionally selected the miRNAs independently of the 3′UTR library to obtain unbiased insights. The relation to PD was the only common selection criterion between the miRNAs and the genes. We first cloned the miRNAs into the expression plasmid pSG5 and verified the constructs by Sanger sequencing. We next transfected the miRNA expression plasmids into 293 T cells. While Northern blot validation for miR-133b has been previously described^19^, we demonstrated the correct miRNA processing and ectopic expression of miR-129-5p, miR-129-1-3p, and miR-873-5p in 293T cells (Fig. 1b–d). Because the miRNAs and genes were selected independently of each other, we identified potential targets of the four miRNAs in the 3′UTR library of the PD-associated genes. In silico consensus target prediction revealed 8069 potential target genes for miR-129-5p (Supplementary Table 2), 3844 for miR-129-1-3p (Supplementary Table 3), 3845 for miR-133b (Supplementary Table 4), and 6620 for miR-873-5p (Supplementary Table 5). Out of the PD-specific 3′UTR library, we subsequently extracted those plasmids that harbored a canonical miRNA binding site for the respective miRNA by sequence alignment of the 3′UTR sequences with the canonical binding sites (8-mer, 7-mer-m8, 7-mer- A1, 6-mer). As a result, we identified 320 3′UTR subregions with binding sites for miR-129-5p, 95 3′UTR subregions for miR-129-1-3p, 100 3′UTR subregions for miR-133b, and 190 3′UTR subregions for miR-873-5p (Supplementary Table 6). As mentioned, we split the 3′UTRs into 1280 overlapping fragments to account for length restrictions and obtain optimal results. In sum, 705 potential MTIs remained and were tested by our semiautomated reporter assay: 140 genes predicted for miR-129-5p were represented by 320 3′UTR reporter constructs, 60 genes predicted for miR-129-1-3p were represented by 95 3′UTR reporter constructs, 69 genes predicted for miR-133b were represented by 100 3′UTR reporter constructs, and 102 genes predicted for miR-873-5p were represented by 190 3′UTR reporter constructs (Fig. 1a).

The target gene 3′UTR reporter constructs harboring more than one exclusive miRNA binding site for one of the four tested miRNAs are summarized in Supplementary Table 7, and the overlap between the target gene 3′UTR constructs for these miRNAs is visualized in Supplementary Fig. 3. We identified 22 target gene 3′UTR vectors with binding sites for miR-873-5p and miR-133b, 6 for miR-133b and miR-129-1-3p, 25 for miR-133b and miR-129-5p, 5 for miR-129-1-3p and miR-129-5p, 14 for miR-873-5p and miR-129-1-3p, and 43 for miR-873-5p and miR-129-5p. A few of the target gene 3′UTR constructs harbored binding sites for 3 different miRNAs: 4 constructs harbored binding sites for miR-873-5p, miR-133b and miR-129-1-3p; 1 construct harbored binding sites for miR-133b, miR-129-1-3p and miR-129-5p; 3 constructs harbored binding sites for miR-873-5p, miR-133b and miR-129-5p; and 1 construct harbored binding sites for miR-873-5p, miR-129-1-3p, and miR-129-5p.

### The semiautomated reporter assay validated up to 80% of the MTIs for miR-129-1-3p

**Fig. 2:**
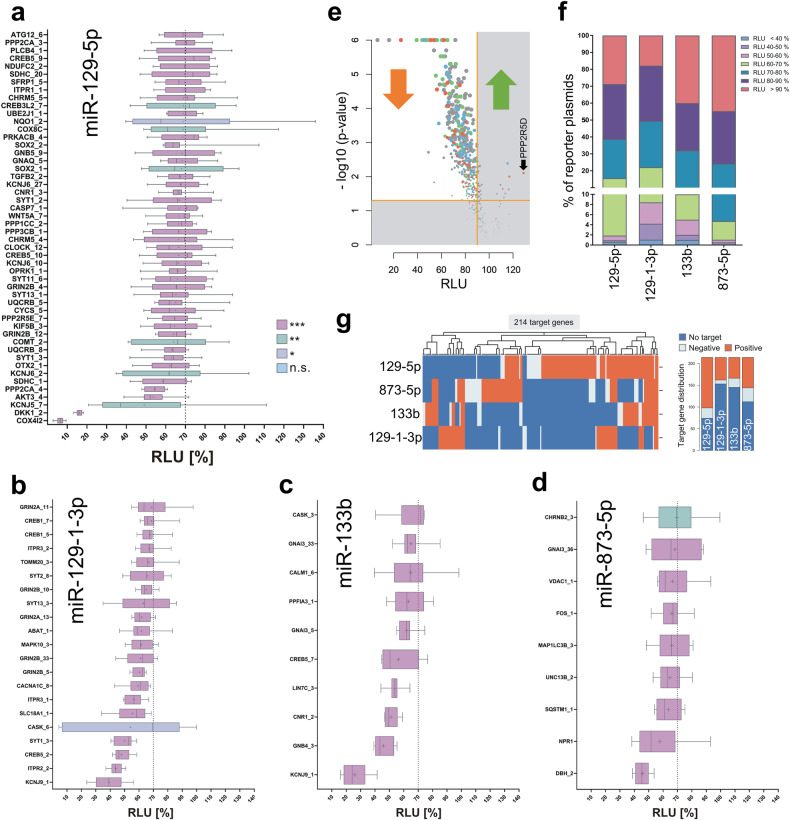
Analyzing the target efficacy of the PD-specific library. **a** Reporter assay results for miR-129-5p. The extracted reporter plasmids corresponding to the predicted target genes of the four PD-associated miRNAs were analyzed for miRNA targeting ability via semiautomated HiTmIR assays. First, 293T cells were cotransfected with the respective miRNA expression plasmid and different reporter plasmids. After 48 h, the cells were lysed, and luciferase activity was determined. The RLU of the respective reporter plasmid was normalized to that of the corresponding cotransfected miRNA with the empty vector pMIR-RNL-TK. The reporter plasmids with a detected RLU <70% are shown. LiMTaR was performed four times in technical duplicates. The colors of the single bars represent the corresponding *p* values. **b** Reporter assay results for miR-129-1-3p. **c** Reporter assay results for miR-133b. **d** Reporter assay results for miR-873-5p. **e** RLUs of all MTIs for all miRNAs measured by the reporter assay versus the negative decade logarithm of the *p* value. The colors represent the four miRNAs (blue: miR-133b; red: miR-873-5p; green: miR-129-5p; gray: miR-129-1-3p). All points in the upper left corner met our criterion of a reduction in gene activity at a significant level. **f** Overall distribution of miRNA target regulation. The analyzed reporter plasmids were categorized by the detected RLU. **g** Heatmap showing the targeting results as an adjacency matrix. Dark blue cells are sets of genes and miRNAs for which no computational evidence exists and for which information has not been obtained. Light blue represents negative validation results (false-positive predictions), and red represents verified interactions.

To experimentally validate the MTIs between the in silico-identified UTRs of the library and the four miRNAs, we performed our semiautomated HiTmIR assays for all 705 predicted MTIs. Following cotransfection of a miRNA with the reporter plasmid, the assay indicated functional binding of the tested miRNAs to the respective 3′UTR as a reduction in RLUs. We distributed the 705 MTIs along with positive and negative controls on 35.25 96-well plates. Each dual luciferase assay was then performed four times, resulting in 35.25 × 4 × 96 = 13,536 single transfections (Fig. 2a–d). The validated MTIs are shown in the upper left quadrant of a volcano plot (Fig. 2g). In line with the original miRATBase criteria, we required a mean reduction to <90% of the relative light units (RLU) compared to the empty miRNA expression vector. As a second criterion, we required a significant nominal p value at an alpha level of 0.05. Notably, the results were presented as the maximal reduction and the maximal significance of all 3′UTR sequences representing that gene. Interestingly, the four miRNAs varied significantly with respect to the number of targeted 3′UTRs and the target effect size (Fig. 2f, g). Out of a total of 320 predicted and tested 3′UTR constructs (i.e., predicted and tested MTIs), 215 were suppressed by miR-129-5p (i.e., verified MTIs). Out of 95 predicted and tested 3′UTR constructs (i.e., predicted and tested MTIs), 76 were suppressed by miR-129-1-3p (i.e., verified MTIs). Out of 190 predicted and tested 3′UTR constructs (i.e., predicted and tested MTIs), 96 were suppressed by miR-873-5p (i.e., verified MTIs). Out of 100 predicted and tested 3′UTR constructs (i.e., predicted and tested MTIs), 55 were suppressed by miR-133 (i.e., verified MTIs). Among these, the validation rate was highest for miR-129-1-3p (80%), while the lowest rate was 50.5% for miR-873-5p.

To understand whether these patterns persist when focusing on stronger reductions, we repeated the consideration with a reduction to less than 70% of the original intensity. Among the 320 3′UTR subregions with binding sites for miR-129-5p, we observed 50 respective reporter plasmids. The original expression of the target gene COX4I2 (cytochrome c oxidase subunit 4I2) was reduced by 6.4% by miR-129-5p, representing the most powerful targeting effect (*p* < 10-6). For miR-129-1-3p, we detected a highly significant reduction in RLUs (*p* < 0.001) for most reporter plasmids. Of the 95 3′UTR sequences for that miRNA, 21 reporter plasmids exhibited a significant RLU reduction to less than 70% of their original expression. The expression of KCNJ9 (potassium inwardly rectifying channel subfamily J member 9) was reduced the most (39.2% reduction). For miR-133b (10 UTRs) and miR-873-5p (9 UTRs) less robust targeting effects were observed. miR-133b decreased the original expression of the target gene KCNJ9 by 26.0%, which was the lowest overall reduction. For miR-873-5p, 45.7% of the RLU of the 3′UTR of the target gene dopamine beta-hydroxylase_2 (DBH_2) remained. The lower number of target genes of miR-133b and miR-873-5p compared to miR-129-5p and miR-129-1-3p is indicative of the differences in the functional abilities of the respective miRNAs. Remarkably, we also identified few genes with a significant upregulation in the target validation (see also the “right” arm in the volcano plot, Fig. 2e), contrasting the expected patterns of reduced activity. The targeting of the PPP2R5D gene (protein phosphatase 2 regulatory subunit B′delta) by miR-133b increased its activity by 130%, indicating a significantly increased expression level (*p* = 0.008). There is increasing evidence that miRNAs can also act as positive regulators of target genes. Recent studies have reported that miRNAs can positively regulate their target genes by binding to the promoter sequences of their respective target genes in the nucleus^36,37^. However, the mechanisms by which a miRNA induces the transcription of a target gene are not fully understood.

**Fig. 3:**
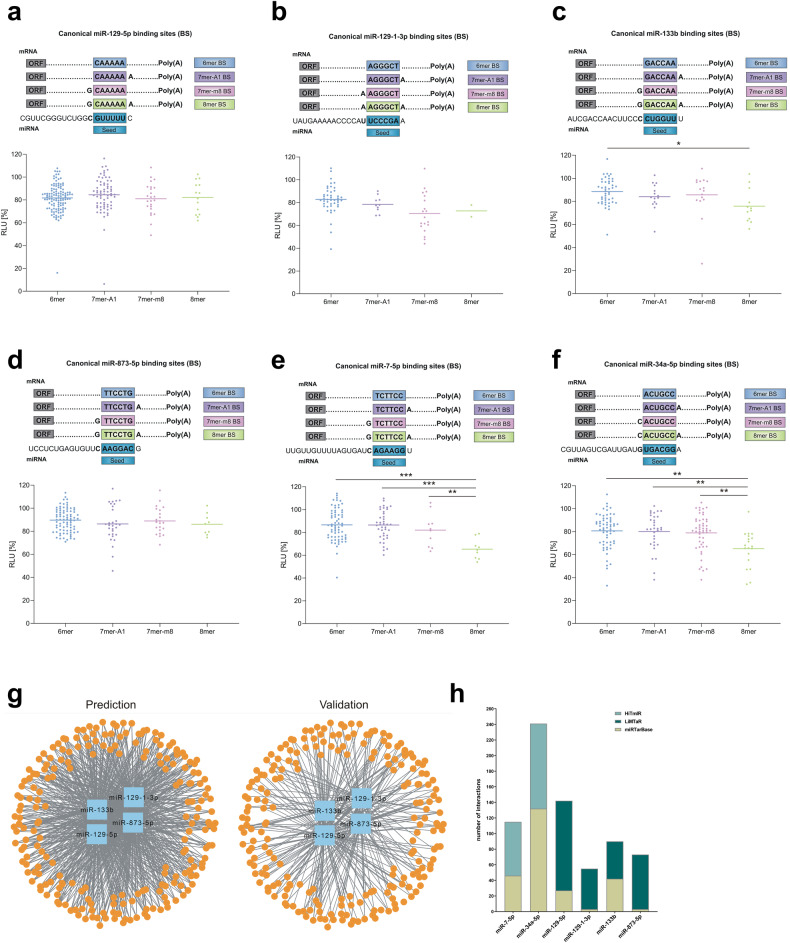
Impact of the miRNA binding sites within a reporter plasmid on miRNA-dependent regulation. We categorized the results by the type of corresponding miRNA binding site within the 3′UTR sequence that was correlated with the respective RLU. The sequences of the different miRNA binding sites for each miRNA are depicted at the top of each subpanel. **a** The 3′UTR sequence of miR-129-5p. **b** The 3′UTR sequences of miR-129-1-3p. **c** The 3′UTR sequences of miR-133b. **d** The 3′UTR sequence of miR-873-5p. **e** The 3′UTR sequence of miR-7-5p. **f** The 3′UTR sequence of miR-34a-5p. **g** Graph-based validation. Left panel: Complete target network. The four miRNAs are presented in the middle as blue nodes, and the target genes are presented as smaller organ nodes. Right panel: validation of the target gene network. All edges without experimental evidence from the previous graph were removed. **h** Comparison of miRNA‒target gene interactions in miRTaRBase versus positive miRNA‒target gene interactions in miRATBase for miR-7-5p, miR-34a- 5p, miR-129-5p, miR-129-1-3p, miR-133b and miR-873-5p. HiTmIR= high-throughput miRNA interaction reporter assay, LIMTaR= library-based miRNA target gene reporter assay (this study), and miRTarBase=microRNA‒target interaction database.

The astonishing variability of the results motivated a more detailed look at the positive and negative binding events for MTIs. In the following section, we address the questions of whether and to what extent the binding site, (i.e., the number or the composition of the complementary nucleotides between UTR sequences and miRNAs), contributes to the variation that we found for the tested miRNAs. For the standardized validation experiments, we considered the 3′UTRs based on the binding site type (6-mer, 7-mer-A1, 7-mer-m8, 8-mer) and the number of miRNA binding sites. Generally, we expect a greater reduction in the RLU for 7-mer and 8-mer binding sites than for 6-mer binding sites. For this analysis, we additionally included data on the targets miR-7-5p and -34a-5p from our previous study; these data were also obtained with our standardized high-throughput miRNA interaction reporter assay (HiTmIR)^23^. For miR-129-5p, the most prominent reduction was detected for the 7-mer-m8 binding site (Fig. 3a). Surprisingly, we found 7-mer and 8-mer binding sites within the 3′UTR sequence of this miRNA that did not reduce the RLU of the target gene. Similarly, we found a limited influence of the binding site type on the RLU for miR-129-1-3p (Fig. 3b). In this case, a 6-mer binding site yielded the most reduced RLU in the target 3′UTR. For miR-133b, we detected a significant decrease in the RLU at the 8-mer sites compared to the 6-mer sites (Fig. 3c). For miR-873-5p, we did not detect a significant difference between the target site types. The 7-mer-A1 target site yielded the strongest RLU reduction (Fig. 3d). After reanalyzing the data from our previous study on miR-7-5p (Fig. 3e) and miR-34a-5p (Fig. 3f), we found a significant negative correlation between the number of binding nucleotides and the reduction in the RLU. In particular, the presence of an 8-mer binding site resulted in a significant reduction compared to that of the other binding sites. The GC content of the respective seed sequences ranged from 16.7% in the seed region of miR-129-5p to 66.7% in the seed regions of miR-129-1-3p and miR-34a-5p and was not related to the correlation between binding and RLU reduction. Notably, for reporter plasmids containing more than one miRNA binding site, we did not observe a significant correlation between the number of miRNA binding sites and the reduction in RLU (Supplementary Fig. 4).

Finally, we summarized the MTI results from the UTR level to the gene level based on the above criteria. We identified a total of 214 potential target genes for at least one of the tested miRNAs (miR-129-5p, miR-129-1-3p, miR-133b, and miR-873-5p; Supplementary Table 8). Considering the MTIs at the gene level as edges in a bipartite graph with 214 genes potentially targeted by at least one miRNA of the four miRNAs results in 214 × 4 = 856 edges (Fig. 3g). Overall, we provide computational evidence for 372 of the 856 edges (43.5%). Based on our experimental data, we considered a gene to be repressed if one of its UTR-based MTIs showed a significant reduction to at most 90%. By analyzing the experimental results of the 372 edges with positive computational evidence, we reached a confirmation rate of 76.9% (i.e., we validated 286 target genes). Overall, 286 of the 856 edges (33.4%) in the bipartite graph had computational and experimental target evidence. Three genes (CXCL12 (C-X-C motif chemokine ligand 12), RAB3B (RAB3B, a member of the RAS oncogene family), and SYT1 (synaptotagmin 1)) were targets of all four miRNAs. There were 23 additional genes that were targets of 3 of the four miRNAs and 52 genes that were targets of two of the four miRNAs. In total, 101 genes were targeted by exactly one miRNA, and no experimental evidence could be found for 35 of the original 214 target genes.

To put the results in the context of currently available targetomes, we compared the number of MTIs to the number of formerly validated MTIs deposited in miRTarBase. Compared to previously published MTIs entered in miRTarBase, we extended the number of MTIs validated by a reporter assay for each miRNA. Including the results of our previous study, we increased the number of validated target gene interactions from 46 to 115 for miR-7-5p (≙2.5-fold), from 132 to 244 for miR-34a-5p (≙1.8-fold), from 27 to 142 for miR-129-5p (≙10.1-fold), from 3 to 55 for miR-129-1-3p (≙18.3-fold), from 42 to 90 for miR-133b (≙2.1-fold), and from 3 to 73 for miR-873-5p (≙24.3-fold) (Fig. 3h). Notably, the opposite comparison demonstrated that none of the MTIs being negative in our assay has been found positive in the miRTarBase.

### Computational analysis highlights the limited influence of the predicted local secondary structure

One of the original goals was to develop a resource that facilitates improved target prediction by providing standardized reporter assay results. In the last section, we described the effects of the target sites on the miRNA targeting efficacy. One piece of information that is not included in most target predictors is the local secondary structure of the 3′UTR, not to mention more complex information such as the tertiary structure. However, RNA structures are known to generate natural cooperation between single-stranded RNA-binding proteins and 3′UTRs^38^. Not surprisingly, the secondary structure seems to affect the binding of specific genes, as demonstrated for miR-159 in plants^39^. Our standardized dataset allowed us to test this hypothesis at a larger scale. While we acknowledge that the cloned 3′UTRs do not necessarily reflect the physiological situation (this remains one of the primary challenges of reporter assays), the cloned sequences and the predictions made from the same sequences are consistent.

**Fig. 4:**
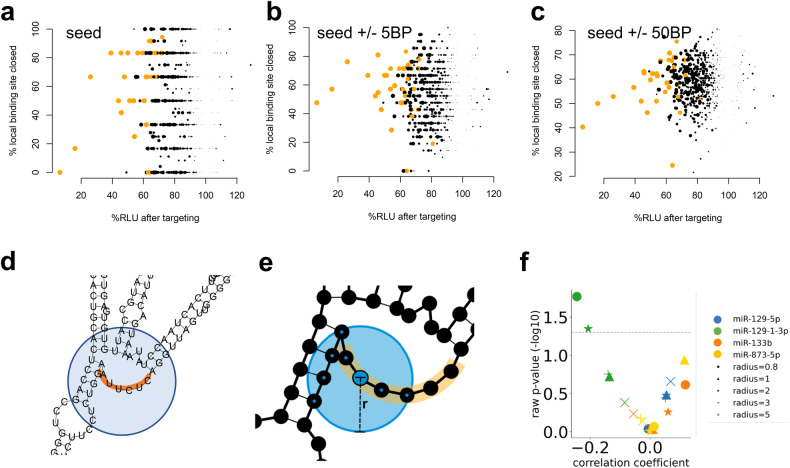
Correlation of the local secondary structure with the repression of target genes. **a** The scatter plot shows the percentage of closed bases that are bound (y-axis) against the reduction in the RLU in %. The expected pattern is a positive correlation: the more closed a binding site is, the lower the reduction in repression should be. The orange dots represent significantly (*p* value < 10^−5^) reduced target sites. **b** The results when the binding site was extended by a window of 5 bases in the 3′ and 5′ directions. **c** The results when the binding site was extended by a window of 50 bases in the 3′ and 5′ directions. **d** Schematic drawing of an example seed region (highlighted in orange) in a secondary structure. The circle in blue shows the area around the binding site. **e** For each base in the binding site, a circle with variable radius (r) was used to determine the number of nucleotides in proximity (bases in the circle are black circles with blue dots). We tested values of 0.8–5 length units for the radius to compute a coverage score for the binding site. **f** Scatter plot describing the correlation between the coverage score and the RLU (*x*-axis) and the respective significance value (*y*-axis). The analysis was performed for each mina (colors) with different radii (shapes). For miR-129-1-3p, we observed a pattern corresponding to the original hypothesis, however, the other miRNAs did not display this pattern.

Thus, sites that are less accessible in the 3′UTR due to folding onto other sites in the same 3′UTR might impair the targeting process. We thus compared the fraction of the seed binding region within the 3′UTR that was paired with the reduction in the RLU. We did not detect a significant correlation for any of the miRNAs (Fig. 4a), indicating that the local folding structure has a limited influence on the reporter assays. By extending the consideration to 5- (Fig. 4b) and 50-base windows (Fig. 4c) to the 3′ and 5′ end of the 3′UTR, we identified a tendency toward a significant reduction in the number of MTIs (*p* < 10^−5^). The latter aspect indicates that while the local secondary structure of the 3′UTR seems to have a limited influence, the overall secondary, and even the tertiary structure, may have an influence.

This investigation requires a more precise model of the region surrounding each binding site. We implemented a score that represents the region surrounding the binding site (Fig. 4d). More precisely, we defined a circle with a variable diameter surrounding each nucleotide in the seed region of the secondary structure and counted the bases within this circle (Fig. 4e). The score was calculated by adding up the counts for each seed base and normalized by the number of bases in the seed. A high score reflected a rather closed secondary structure, while a low score reflected an open structure. By testing the radii between 0.8 and 6 length units, we identified patterns that differed significantly between miRNAs. Depending on the miRNA, we observed weak positive correlations between the coverage score and the RLU (which reflects the original assumption of better binding when the binding site is open) but also significant negative correlations (Fig. 4f). For miR-129-1-3p, we identified significant negative correlations between the coverage score and the RLU. These examples highlight that developing a general model for improved targeting of miRNAs via secondary structure is challenging and that factors beyond the 3′UTR secondary structure also have to be considered. A tool to visualize the binding sites of one or multiple miRNAs within a UTR is freely available (https://ccb-web.cs.uni-saarland.de/utr-seremato).

### Effect of miRNA overexpression on endogenous protein expression

**Fig. 5:**
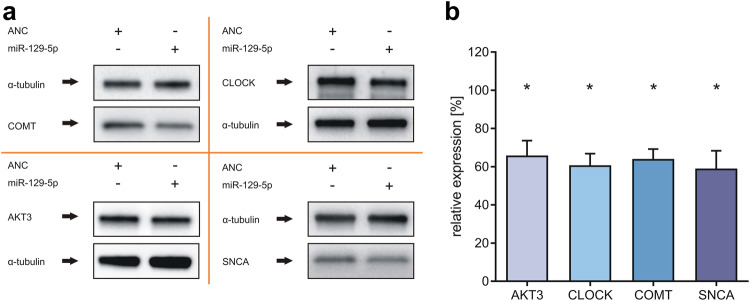
Western blot analysis of miR-129-5p. SH-SY5Y cells were transfected with either ANC or the miRNA mimic. After 48 h, the changes in the protein expression of AKT3, CLOCK, COMT, and SNCA induced by miR-129-5p were determined using specific antibodies against these proteins. Western blot analysis for each protein was performed for three independent replicates. **a** Results of the analysis of the regulation of SNCA, CLOCK, AKT3, and COMT protein expression by miR-129-5p. **b** Quantitative analysis of the changes in the protein expression of AKT3, CLOCK, COMT, and SNCA induced by miR-129-5p are shown in the bar chart (mean ± standard deviation). Asterisks indicate a significant reduction.

To complement the expression analysis of miRNAs determined by the dual luciferase assays with both exogenous reporter plasmids and exogenous miRNA expression plasmids, we analyzed the effect of exogenous miRNA on endogenous proteins by Western blotting. For validation by Western blotting, we selected miR-129-5p, which has been previously associated with PD^23^. We selected proteins that have also been previously associated with PD and were identified as potential mRNA targets of miR-129-5p by our reporter assays. The neuronal cell line SH-SY5Y was transfected with the respective miRNA mimics, and RT‒qPCR was used to evaluate the ectopic expression of miR-129-5p (Supplementary Fig. 2). Ectopic expression of miR-129-5p reduced the expression of AKT3 (AKT serine/threonine kinase 3) to 65.8%, CLOCK (clock circadian regulator) to 60.7%, COMT (catechol-O-methyltransferase) to 64%, and SNCA (α-synuclein) to 59% (Fig. 5a, b). In summary, Western blot analysis confirmed the MTIs observed by dual luciferase assays. This finding agrees with our previous findings, which confirmed the results of dual luciferase assays and Western blot analyses^17,23,40–42^.

## Discussion

Understanding the interactions between miRNAs and target genes is central to the characterization of molecular signaling cascades, including signaling cascades, in a disease context. As mentioned above, there are more than 44,571,700 potential miRNA target interactions (MTIs). Computational approaches are certainly the first choice for tackling the task of elucidating complex networks. Ultimately, however, these approaches require experimental approaches to verify the predicted MTIs. We employed a systematic approach for MTI determination under standardized conditions by using a 3′UTR library enriched for genes associated with Parkinson’s disease. We analyzed this library by more than 13,000 single transfections for a set of four miRNAs that have been previously associated with PD. Mature miR-129-5p and miR-129-1-3p, which are both processed from the same precursor, premir-129, were significantly upregulated after the induction of a PD-like phenotype in LUHMES cells, as shown in our previous study^23^. A study by Kim et al. showed that the precursor premiR-129-2, which can also be processed into the mature miR-129-5p, was enriched in the midbrain of PD patients^33^. The same study showed a significant reduction in the expression of the mir-133b precursor. A significantly reduced miR-133b level was also demonstrated in the plasma of PD patients and in different models mimicking a PD-like phenotype^33,43–45^. miR-873-5p has been previously described to be neuroprotective in a neuroinflammatory model of PD^34^.

Depending on the tested miRNA, differences were detected in the overall RLU value distribution, the number of significant target gene 3′UTR reporter plasmids and the positive rate. We found that the effectiveness of miRNAs strongly varies between distinct signaling pathways. Especially for miR-873-5p, most reporter plasmids showed only a slight reduction in the RLU, a small number of significant target genes and a low positive rate compared to those of the other tested miRNAs. Although miRNAs that regulate only small numbers of target genes in the dopamine and PD-associated pathways might be less relevant for these signaling pathways, these miRNAs might nevertheless impact the pathogenesis of the disease via other signaling pathways. The miR-873-5p, which has been primarily described as neuroprotective in the context of neuroinflammation, may regulate target genes directly associated with the PD-associated immune response^34^. Although our study focused on the generation of miRNA‒target gene networks in affected dopaminergic neurons, identifying target genes of miR-873-5p that are associated with the immune system could further help to elucidate the role of this miRNA in PD pathogenesis, especially in the context of neuroinflammation.

While previous high-throughput studies on the impact of miRNA binding often did not exclude indirect effects^46,47^, our high-throughput analysis focused on direct interactions between a miRNA binding site and the respective miRNA. The results of the analysis of the binding sites of miR-34a-5p, miR-7-5p, miR-129-1-3p and miR-133b were comparable to those of other miRNAs^46,47^. The higher the number of potential binding nucleotides within a 3′UTR was, the greater the effect of miRNA-induced regulation was. This, however, did not apply to reporter plasmids containing a 7-mer-m8 binding site that showed a low or no reduction in the RLU. In contrast, no significant effect between the different types of binding sites was detected for the miRNAs miR-129-5p and miR-873-5p. Several additional factors may contribute to MTIs, including the surrounding sequence in the 3′UTR of the target gene and the secondary structure of the mRNA^47–50^. The latter hypothesis is supported by in silico predictions of the local secondary structure of mRNAs around the miRNA binding site, indicating reduced accessibility of the miRNA binding site for genes with high RLUs.

Independent of the mRNA secondary structure, the mere flanking sequences, for example, 3′ complementarity, may also influence the MTI as well as the nucleotide composition of the miRNA seed region^51^. Of particular interest in this context are the differences in the GC content ranging from 16.7% in the seed region of miR-129-5p to 66.7% in the seed regions of miRNAs miR-34a-5p and miR-129-1-3p. Notably, miRNAs with a low GC content in the seed region were associated with noncanonical targeting^51^. In addition to the analysis of specific MTIs, MTI networks that are largely predicted only by in silico tools await further identification and validation via experimental approaches. As recently shown, target sites within mRNAs can act cooperatively, resulting in greater repression of the target mRNA than that caused by independent action at each site^52^. Similarly, the number of miRNAs, the number of targets, and the availability of free RISCs are important, as each plays a role in the development and status of MTI networks.

The functional assays that are required to contribute to MTI network analyses have severe limitations and biases. To analyze the interactive effects of two or more miRNAs, transfection assays can be used, but they do not allow us to define the stoichiometric relationships between the miRNAs and their target MTIs. The interpretability of cotransfection assays is further impacted by the variable and/or frequently low transfection efficacy. Furthermore, the cellular background can also affect the functionality of MTIs^53^. Different cell types express varying amounts of different RNA-binding proteins (RBPs)^54^, which can affect the ability of a miRNA to regulate target genes. RBPs can modulate the secondary structure of target 3′UTRs, enhancing the binding capability of miRNAs^55^. On the other hand, RBPs binding to target 3′UTRs can prevent miRNAs from binding by masking their binding motifs. This can occur when RBPs bind to the same site as the miRNA or when they bind to a nearby site that overlaps with the miRNA binding site^56,57^.

Independent of these challenges, the presented systematic 3′UTR library-based approach for targetome determination provides a basis for subsequent MTI network analyses. To acknowledge the complexity of this scenario, we employed a standardized library-based functional approach with 13,536 single transfections and identified a very dense MTI network with 447 MTIs for PD. Our approach to studying MTIs in a disease context can serve as a foundation to experimentally validate millions of potential MTIs, especially in specific disease phenotypes. From our study, we derive the following concrete scenarios and next steps: (1) Standardization of reporter assay experiments is essential for obtaining sufficient high-quality data and training artificial intelligence approaches to improve the prediction of targets. (2) It is mandatory to perform standardized experiments at the same scale for cooperative binding scenarios. Only with the respective datasets paired with improved computational models can the effects and side effects of miRNA-mediated therapies be modeled, facilitating miRNA therapeutic applications.

### Supplementary information

Supplementary Information

Supplementary Table 1

Supplementary Table 2

Supplementary Table 3

Supplementary Table 4

Supplementary Table 5

Supplementary Table 6

Supplementary Table 7

Supplementary Table 8
