# Supplementary material for: Experimental capture of miRNA targetomes: disease-specific 3′UTR library-based miRNA targetomics for Parkinson’s disease
Source: Exp Mol Med. 2024 Apr 1;56(4):935–45. doi: 10.1038/s12276-024-01202-5 (PMC11059366; doi:10.1038/s12276-024-01202-5)
Supplement: Supplementary file 1 — Supplementary Information [file 12276_2024_1202_MOESM1_ESM.pdf]

Supplementary Figure 1

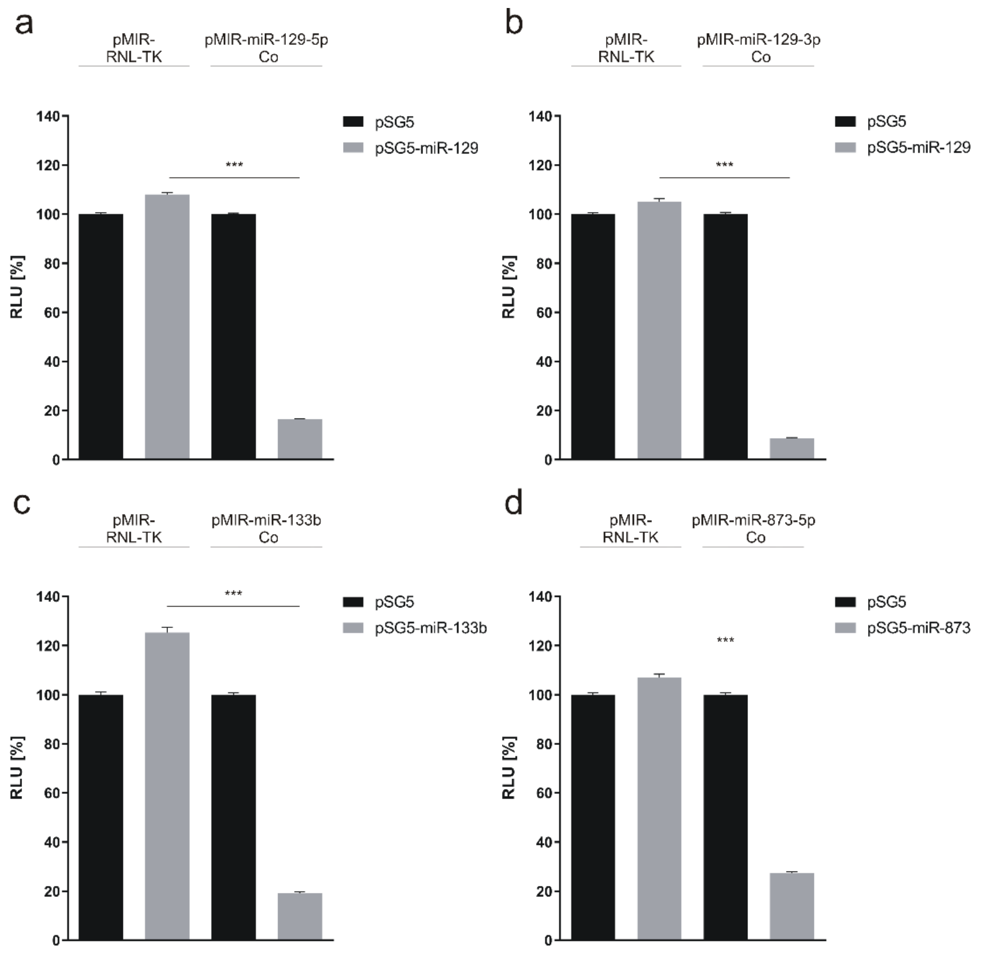

Supplementary Figure 1: Results of the library based MiRNA Target gene Reporter-Assay controls. Empty expression plasmids and reporter plasmids as well as positive controls and miRNA expression plasmids for miR-129-5p **(a)**, miR-129-1-3p **(b)**, miR-133b **(c)**, and miR-873-5p **(d)** were transfected in 293T cells in the indicated combinations and analyzed by LiMTaR.

Supplementary Figure 2

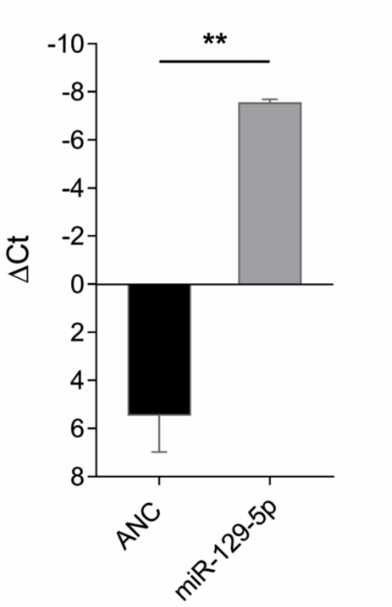

Supplementary Figure 2: Determination of miR-129-5p expression in SH-SY5Y cells by qRT-PCR. SH-SY5Y cells were transfected with either ANC, or miR-129-5p Mimic. After 48h, RNA was isolated, and expression was determined using specific primers for miR-129-5p. RNU6B served as endogenous control. Data represent three independent replicates in technical duplicates.

Supplementary Figure 3

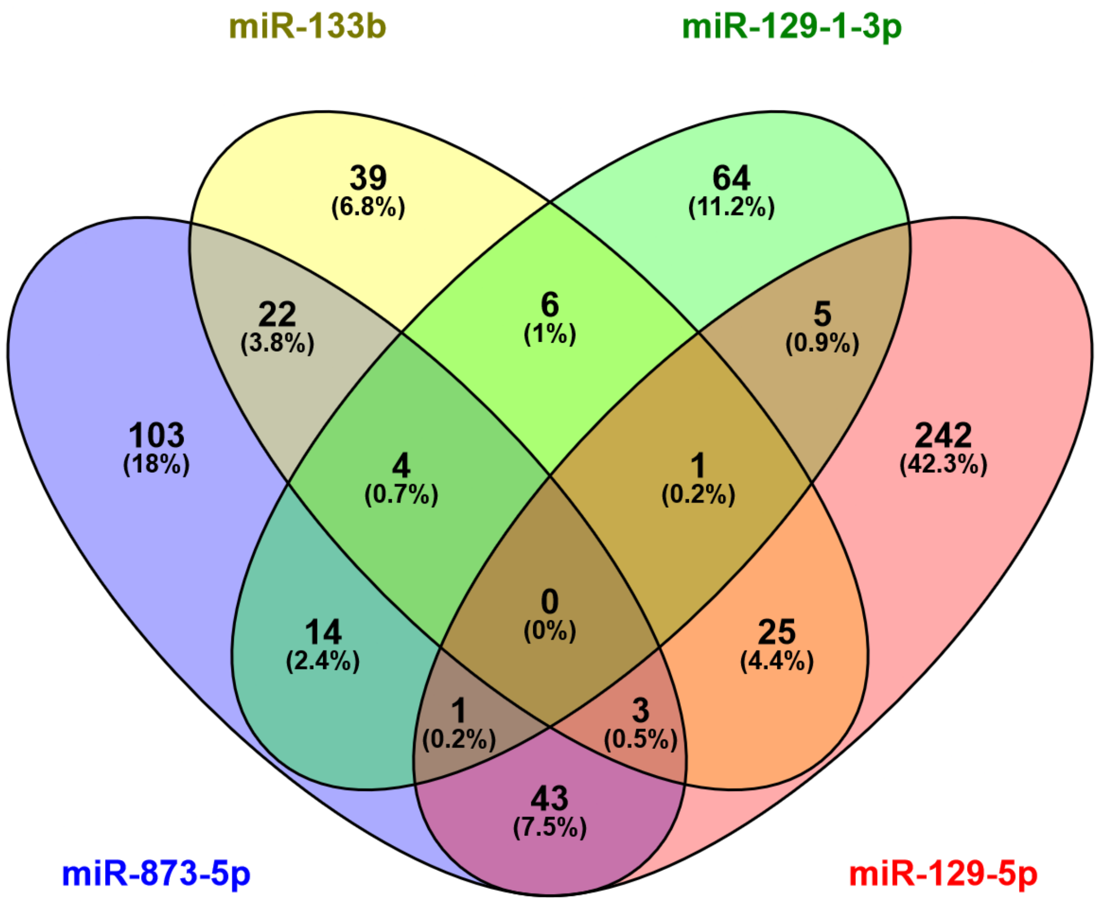

Supplementary Figure 3: Venn diagramm of the Target gene 3'UTR constructs harboring more than one exclusive miRNA binding site of one of the four tested miRNAs

Supplementary Figure 4

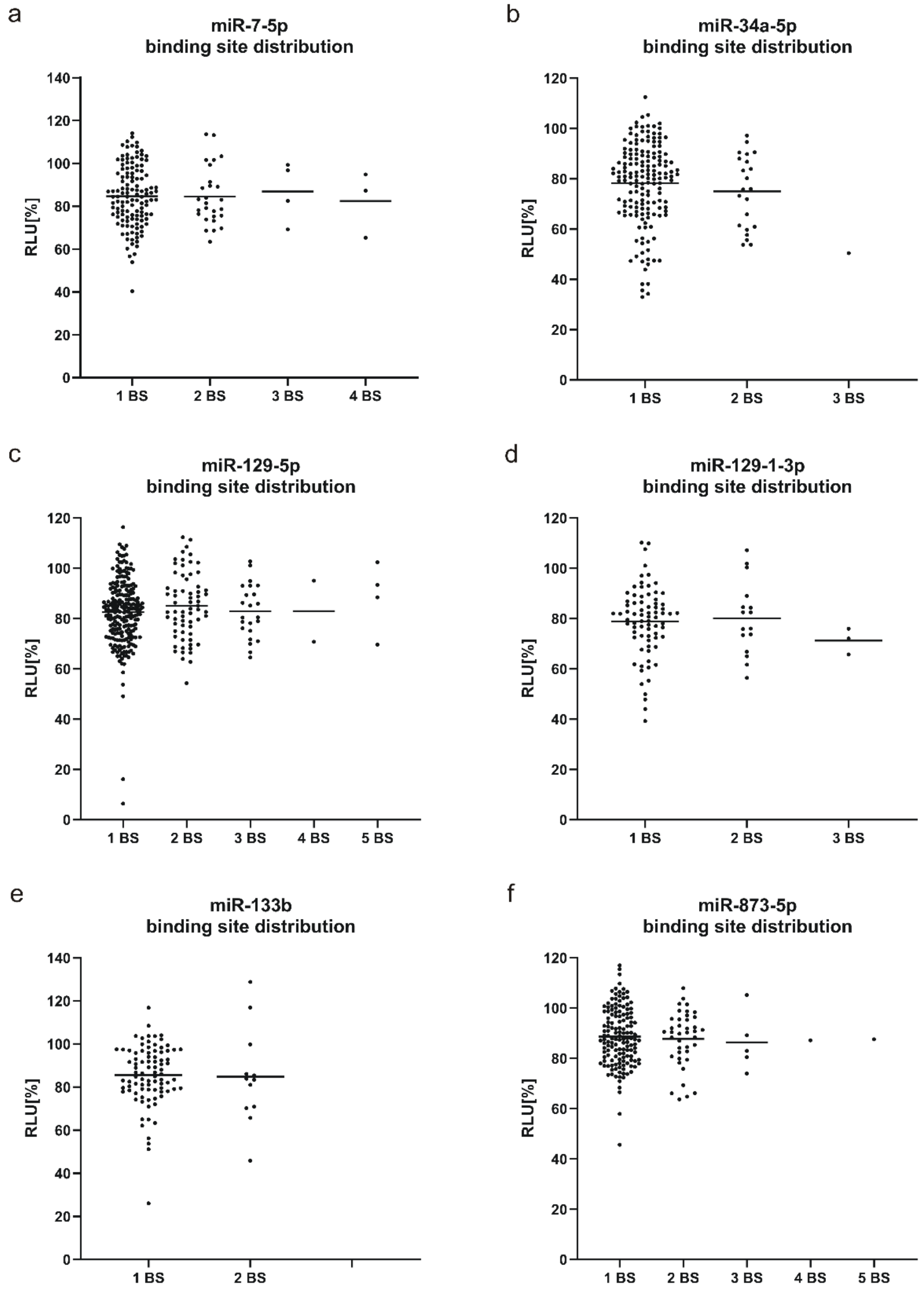

Supplementary Figure 4: Impact of the number of miRNA binding sites within a reporter plasmid on detected miRNA-caused regulation. The 3'UTR sequences tested with miR-7-5p **(a)**, miR-34a-5p **(b)**, miR-129-5p **(c)**, miR-129-1-3p **(d)**, miR-133b **(e)**, and miR-873-5p **(f)** were categorized by the number of corresponding miRNA binding sites within the 3'UTR sequences and correlated to the respective RLU.
